# Evaluation and optimisation of direct transesterification methods for the assessment of lipid accumulation in oleaginous filamentous fungi

**DOI:** 10.1186/s12934-021-01542-1

**Published:** 2021-03-03

**Authors:** Anne Marie Langseter, Simona Dzurendova, Volha Shapaval, Achim Kohler, Dag Ekeberg, Boris Zimmermann

**Affiliations:** 1 Faculty of Science and Technology, Norwegian University of Life Sciences, Postbox 5003, 1432 Ås, Norway; 2 Faculty of Chemistry, Biotechnology and Food Science, Norwegian University of Life Sciences, P.O. Box 5003, 1432 Ås, Norway

**Keywords:** Oleaginous microorganisms, Biodiesel, Biofuel, Methyl esters, In situ transesterification

## Abstract

**Background:**

Oleaginous filamentous fungi can accumulate large amount of cellular lipids and potentially serve as a major source of oleochemicals for food, feed, chemical, pharmaceutical, and transport industries. Transesterification of microbial oils is an essential step in microbial lipid production at both laboratory and industrial scale. Direct transesterification can considerably reduce costs, increase sample throughput and improve lipid yields (in particular fatty acid methyl esters, FAMEs). There is a need for the assessment of the direct transesterification methods on a biomass of filamentous fungi due to their unique properties, specifically resilient cell wall and wide range of lipid content and composition. In this study we have evaluated and optimised three common direct transesterification methods and assessed their suitability for processing of fungal biomass.

**Results:**

The methods, based on hydrochloric acid (Lewis method), sulphuric acid (Wahlen method), and acetyl chloride (Lepage method), were evaluated on six different strains of Mucoromycota fungi by using different internal standards for gas chromatography measurements. Moreover, Fourier transform infrared (FTIR) spectroscopy was used for the detection of residual lipids in the biomass after the transesterification reaction/extraction, while transesterification efficiency was evaluated by nuclear magnetic resonance spectroscopy. The results show that the majority of lipids, in particular triglycerides, were extracted for all methods, though several methods had substandard transesterification yields. Lewis method, optimised with respect to solvent to co-solvent ratio and reaction time, as well as Lepage method, offer precise estimate of FAME-based lipids in fungal biomass.

**Conclusions:**

The results show that Lepage and Lewis methods are suitable for lipid analysis of oleaginous filamentous fungi. The significant difference in lipid yields results, obtained by optimised and standard Lewis methods, indicates that some of the previously reported lipid yields for oleaginous filamentous fungi must be corrected upwards. The study demonstrates value of biomass monitoring by FTIR, importance of optimal solvent to co-solvent ratio, as well as careful selection and implementation of internal standards for gas chromatography.

## Introduction

Microbial oils, produced by a range of oleaginous microorganisms, are being considered as one of the primary sources of oleochemicals for food, feed, chemical, pharmaceutical, and transport industries. Oleaginous microorganisms, such as algae, bacteria and fungi, can accumulate cellular oils in amounts above 20% of dry biomass (%w/w), and often in 50–80%w/w range [1, 2]. The cellular oils are mainly produced in the form of free fatty acids and acylglycerols (mostly as triglycerides) and are stored in the globular organelles called lipid bodies. Microbial oils can have similar chemical composition to animal and vegetable oils, ranging from the valuable and highly nutritious omega-3 and omega-6 long-chain polyunsaturated fatty acids (PUFAs), to the biodiesel-compatible monounsaturated and saturated fatty acids (MUFAs and SFAs). Amongst various types of oleaginous microorganisms, Mucoromycota fungi have gained interest due to their versatile metabolism capable to utilize a wide range of feedstock, including waste materials and industrial raw rest materials [3–5]. These filamentous fungi are powerful cell factories able to valorize various feedstocks into a range of marketable products, such as lipids, biopolymers, pigments, proteins, enzymes, and organic acids [6, 7]. Biomass of these oleaginous filamentous fungi can contain large amount of lipids, up to 86% of dry weight [1, 7], which is often significantly higher than in oleaginous plants and single cell phototrophs, such as green algae, diatoms and cyanobacteria [1, 2].

Microbial lipid production at both laboratory and industrial scale includes several upstream and downstream stages, such as cultivation, separation of biomass, cell disruption, oil extraction, and transesterification. At both research and industrial scale, microbial oils are usually extracted by employing various solvents of different polarities [8–10]. However, compared to the plant and animal lipids, extraction of lipids from the microbial biomass is often hindered by the strong and resilient cell wall, which can lead to the reduced extraction performance [11]. Therefore, cell disruption methods are often employed in order to break up the cellular wall and membrane as a precondition for the effective extraction of oils from the microbial biomass. Cell disruption methods include different mechanical and chemical pretreatments, such as bead beating (bead milling), ultrasonication, microwave, enzymes, and acid or base hydrolysis [12, 13]. Recently, we have demonstrated that Fourier transform infrared (FTIR) spectroscopy could be used to evaluate and monitor lipid extraction processes, and to identify cell wall components, such as polyphosphates, polyuronides (polymers of glucuronic acid such as glucuronans), and polyglucosamines (such as chitin and chitosan) biopolymers in Mucoromycota fungi, that may negatively affect the extraction process [14].

Once the extraction of lipids from the microbial biomass is accomplished, lipid yield and chemical composition is analysed. Transesterification is a key step in the lipid analysis since chemical composition of extracted oils is predominantly based on the qualitative and quantitative analysis of methyl esters of fatty acids (FAMEs) due to their volatility and thermal stability. After conversion of lipids to FAMEs, the extracted mixture is routinely analysed by gas chromatography (GC) coupled with various detectors [15, 16]. However, total lipid yield can be estimated by the total FAMEs yield only if the predominant lipid classes present in the biomass can be converted into FAMEs, which is the case for free fatty acids and their derivatives, such as acylglycerols and glycerophospholipids. Moreover, the conversion of acylglycerols and other fatty acid-based lipids into FAMEs is essential for industrial production of biodiesel. Both laboratory and industrial researches are exploring process of direct transesterification, where disruption, extraction and transesterification processes are combined into one operation [13]. Direct transesterification can considerably reduce costs, increase sample throughput and improve yields. There is a number of methods for the direct transesterification of microbial biomass, and they differ mainly by the choice of catalyst, usually either acid or base [10, 15, 17–20], though some methods use both [21, 22]. Compared to the standard two-step extraction-transesterification methods, such as Folch and Bligh and Dyer methods [8, 9], direct transesterification often results in higher FAMEs yields [17, 18, 21]. It should be noted that direct transesterification also includes chemical cell disruption obtained by an acid or base catalyst, and that acid/base hydrolysis of various cell components (other than lipids) can compete with transesterification reaction. Our recent study has demonstrated that acid hydrolysis can significantly increase extraction efficiency during a lipid analysis of filamentous fungal biomass [14]. Moreover, we have recently demonstrated that the combination of microtiter plate-based cultivation, with the direct transesterification monitored by high-throughput FTIR spectroscopy, can be used for high-throughput screening of filamentous fungi and other oleaginous microorganisms for the production of low and high‑value lipids [23–26]. Application of such high-throughput methodology saves valuable time and decreases costs in the development of bioprocesses for both nutraceutical and biofuel industries.

There is a need for the assessment of the direct transesterification methods on a biomass of filamentous fungi due to the unique properties of these oleaginous microorganisms. Specifically, oleaginous filamentous fungi have resilient cell wall made of various biopolymers [27], in particular glucosamines (chitin and chitosan). For example, *Mucor* can have cell wall thickness of 2 µm [28], and both *Mucor* and *Absidia* have been identified as one of the most promising chitin and chitosan producers, with the maximum reported yield of about 35% of dry weight [29, 30]. Another common cell wall biopolymer in Mucoromycota is polyphosphate, which functions as energy storage component and an anion counter-ion for chitin and chitosan [31]. In our previous study, we have found that the presence of these biopolymers hampers extraction of lipids in Mucoromycota biomass by standard lipid analysis methods [14]. Finally, standard direct transesterification methods were developed on biomass with relatively low content of lipids (approx. 5–25% of dry weight), while Mucoromycota can have extraordinary high content of lipids, regularly exceeding 30% of dry weight and reaching over 80% [1, 7]. In general, standard direct transesterification methods were not developed to tackle biomasses with extremely resilient and chemically complex cell wall, and extremely high content of lipids. Although a number of direct transesterification methods have been tested on filamentous fungi, they either failed [32], or were focused on the industrial processes rendering them overly time-consuming for routine research analyses [18, 33–36]. This is unfortunate given that incentive for applying these methods for analysis of filamentous fungi is high, as exemplified by the two recent studies showing clear advantage of direct transesterification methods, compared with standard two-step methods, in routine analysis of fungal biomass [14, 37].

Therefore, the aim of this study was to evaluate and optimise several common direct transesterification methods, and to assess their suitability for processing of fungal biomass of oleaginous Mucoromycota filamentous fungi in screening studies. In addition to fungal biomass, pure vegetable oil (olive oil) was used as a control sample to assess these methods. Several modifications of the three common direct transesterification methods were employed, namely methods based on hydrochloric acid (Lewis method) [10], sulphuric acid (Wahlen method) [19], and acetyl chloride (Lepage method) [38]. The modifications included variations in pretreatments, reaction times, and addition of co-solvents for improved reaction conditions. Different internal standards for GC-FID measurement were used to evaluate the transesterification efficiency and optimise the methods. Moreover, the methods were evaluated by using FTIR spectroscopy for the detection of residual lipids in the biomass after the transesterification reaction. Furthermore, transesterification efficiency of conversion of acylglycerols to FAME was evaluated by nuclear magnetic resonance (NMR) spectroscopy. The methods were compared in terms of the FAMEs yield and fatty acid composition. Finally, the optimised direct transesterification method was demonstrated in a typical high-throughput screening-study workflow, involving microreactor cultivation and routine assessment of biomass lipids by FTIR and GC-FID.

## Materials and methods

### Fungal strains

Six strains of Mucoromycota oleaginous filamentous fungi were used in the study: *Mucor circinelloides* VI 04473, *Umbelopsis vinacea* CCM F539, *Absidia glauca* CCM 451, *Lichtheimia corymbifera* CCM 8077, *Cunninghamella blakesleeana* CCM F705, and *Amylomyces rouxii* CCM F220. Fungi were obtained in agar slants and dishes or in lyophilized form, from the Czech Collection of Microorganisms, Brno, Czech Republic (CCM) and the Norwegian School of Veterinary Science, Oslo, Norway (VI). All the selected oleaginous filamentous fungi were identified as a potentially good fungal lipid producers and were able to accumulate between 23 and 47% of lipids [26].

### Cultivation of fungi in high‐throughput Duetz-MTP screening system

All six fungal strains were cultivated in the Duetz microtiter plate screening (Duetz-MTPS) system (Enzyscreen, Netherlands). Growth was done in two steps-first, growth on standard agar medium for preparing spore inoculum and second, growth in nitrogen limited broth media in Duetz-MTPS. The cultivation was performed in two independent biological replicates for each fungal strain. Biological replicates were prepared on separate MTPS plates and cultivated at different time points for each fungal strain. For every biological replicate, fresh spore suspension was prepared.

For the preparation of spore inoculum, *Umbelopsis vinacea* was cultivated on potato dextrose agar (PDA), while all other strains were cultivated on malt extract agar (MEA). MEA was prepared by dissolving 30 g of malt extract (Merck, Germany), 5 g of peptone (Amresco, USA) and 15 g of agar powder (Alfa Aesar, ThermoFischer, Germany) in 1 l of distilled water and autoclaved at 115 °C for 15 min. PDA was prepared by dissolving 39 g of potato dextrose agar (VWR, Belgium) in 1 l of distilled water and autoclaved at 115 °C for 15 min. Agar cultivation was performed 7 days at 25 °C. Fungal spores were harvested from agar plates with a bacteriological loop after the addition of 10 ml of sterile 0.9% NaCl solution.

The main components of the nitrogen limited broth media were according to the Kavadia et al. [39] with modifications (g l^− 1^) [24]: glucose 80, yeast extract 3, KH_2_PO_4_ 7, Na_2_HPO_4_ 2, MgSO_4_·7H_2_O 1.5, CaCl_2_·2H_2_O 0.1, FeCl_3_·6H_2_O 0.008, ZnSO_4_·7H_2_O 0.001, CoSO_4_·7H_2_O 0.0001, CuSO_4_·5H_2_O 0.0001, MnSO_4_·5H_2_O 0.0001. Media were autoclaved for 15 min at 121 °C. pH of broth media was 6.0 ± 0.3. Cultivation in broth media was performed in Duetz-MTPS, consisting of 24 square polypropylene deep-well microtiter plates, low evaporation sandwich covers and extra high cover clamps, which were placed into the shaker MAXQ 4000 (Thermo Scientific, Germany). 7 ml of sterile broth media was filled into the autoclaved microtiter plates and each well was inoculated with 50 µl of spore inoculum. Cultivation was performed for 7 days at 25 °C and 400 rpm agitation (1.9 cm circular orbit). *Absidia glauca*, *Lichtheimia corymbifera* and *Cunninghamella blakesleeana* were growing in a pellet form, while other strains were growing in a form of dispersed mycelium.

### Cultivation of fungi in Erlenmeyer flasks

In addition to the Duetz-MTPS cultivation, two selected fungi, namely *Mucor circinelloides* and *Umbelopsis vinacea*, were also cultivated in Erlenmeyer flasks. Same as Duetz-MTPS cultivation, flask cultivation was done in two steps-first, growth on standard agar medium for preparing spore inoculum and second, growth in nitrogen limited broth media in Erlenmeyer flasks. 100 ml of sterile broth media (see above) was placed into 500 ml Erlenmeyer flasks and inoculated with 100 µl of abovementioned spore inoculum. Cultivation was performed for 7 days at 25 °C and 130 rpm agitation (2.25 cm circular orbit) in the shaking incubator Climo-Shaker ISF1-X (Kuhner, Germany). Both strains were growing in a form of dispersed mycelium.

### Preparation of biomass

The growth media were separated from the fungal biomass by transferring the fermentation broth with plastic Pasteur pipettes into 15 ml Falcon tubes and the subsequent centrifugation at 13,500 rpm for 15 min at 4 °C. Fungal biomass from Falcon tubes was washed three times with cold distilled water and filtered under vacuum using a Whatman No. I filter paper (GE Whatman, USA). Washed fungal biomass was frozen at − 20 °C and then lyophilized 24 h in a FreeZone 2.5 freeze-dryer (Labconco, USA) at − 50 °C and 0.01 mbar pressure. All samples were stored at − 20 °C until analysis.

### Direct transesterification

We have used three standard transesterification methods. Prior to each transesterification process, the samples were preprocessed by bead beating for cell wall disruption and homogenization. Shortly, the main difference between the transesterification methods is acid catalyst: hydrochloric acid in Lewis method, sulphuric acid in Wahlen method, and acetyl chloride in Lepage method. As an additional difference, Wahlen 1 method was performed in a microwave oven, while all other methods (including Wahlen 2 method) were conducted in a heating block. Finally, all methods were modified by either adding chloroform co-solvent (Lepage 2 method) or by increasing the amount of chloroform co-solvent (Lewis 2 method). The detailed description of the methods is provided below.

The following direct transesterification methods were applied on either fungal biomass from flask cultivations (*Mucor circinelloides* and *Umbelopsis vinacea*) or on pure vegetable (olive) oil. Each sample was processed in triplicate per each direct transesterification method.

#### Hydrochloric acid method 1 (Lewis 1)

Direct transesterification was performed according to Lewis et al. [10] with the modifications: 2 ml screw-cap polypropylene (PP) tube was filled with 30 ± 5 mg freeze dried biomass or vegetable oil, approx. 250 ± 30 mg (710–1180 µm diameter) acid-washed glass beads and 600 µl of methanol. The fungal biomass was homogenized in a Percellys Evolution tissue homogenizer (Bertin Technologies, France) at 5500 rpm, 6 × 20 s cycles. The processed biomass was transferred into glass reaction tube by washing the PP tube with 2400 µl of methanol–chloroform–hydrochloric acid solvent mixture (7.6:1:1v/v) (3 × 800 µl). 1.02 mg of C13:0 TAG internal standard in 100 µl of hexane was added to the glass reaction tube (100 µl from a 10.2 mg/ml^− 1^ glyceryl tridecanoate (C_42_H_80_O_6_, C13:0 TAG (13:0/13:0/13:0), Sigma-Aldrich, USA)). The reaction mixture was incubated at 90 °C for 1 h in a heating block, followed by cooling to room temperature. 0.88 ± 3 mg of C15:1 FAME internal standard in 100 µl of hexane was added to the glass reaction tube (100 µl from a 9.1 mg/ml^− 1^ methyl 10(Z)-pentadecenoate; C_16_H_30_O_2_, C15:1 FAME, Larodan, Sweden), followed by addition of 1 ml of distilled water. FAMEs were extracted by the addition of 2 ml hexane–chloroform (4:1 v/v) followed by 10 s vortex mixing. The reaction tube was centrifuged at 3000 rpm for 5 min at 4 °C, and the upper (organic) phase was collected in glass tube. The hexane–chloroform extraction (extractive workup) was performed thrice. The residual biomass was stored at − 20 °C for FTIR measurements. The solvent in glass tube was evaporated under nitrogen at 30 °C, and small amount of anhydrous sodium sulphate (approx. 5 mg) was added in glass tube. FAMEs were transferred into GC vial by washing the glass tube with 1500 µl hexane (2 × 750 µl) containing 0.01% butylated hydroxytoluene (BHT, Sigma-Aldrich, USA) followed by 5 s vortex mixing.

#### Hydrochloric acid method 2 (Lewis 2)

Direct transesterification was performed according to Lewis et al. [10] with modifications: 2 ml screw-cap PP tube was filled with 30 ± 5 mg freeze dried biomass or vegetable oil, approx. 250 ± 30 mg (710–1180 µm diameter) acid-washed glass beads, and 500 µl of chloroform. 1.02 mg of C13:0 TAG internal standard in 100 µl of hexane was added to the PP tube. The fungal biomass was homogenized in a Percellys Evolution tissue homogenizer at 5500 rpm, 6 × 20 s cycles. The processed biomass was transferred into glass reaction tube by washing the PP tube with 2400 µl of methanol–chloroform–hydrochloric acid solvent mixture (7.6:1:1 v/v) (3 × 800 µl). Finally, 500 µl of methanol was added into glass reaction tube. The reaction mixture was incubated at 90 °C for either 60, 90 or 120 min in a heating block, followed by cooling to room temperature. 0.88 ± 3 mg of C15:1 FAME internal standard in 100 µl of hexane was added to the glass reaction tube, followed by addition of 1 ml of distilled water. The fatty acid methyl esters (FAMEs) were extracted by the addition of 2 ml hexane followed by 10 s vortex mixing. The reaction tube was centrifuged at 3000 rpm for 5 min at 4 °C, and the upper (organic) phase was collected in glass tube. The lower (water phase) was extracted two more times, but now by the addition of 2 ml hexane–chloroform mixture (4:1 v/v). The residual biomass was stored at − 20 °C for FTIR measurements. The organic phase in the glass tube was dried and prepared for the GC measurement according to Hydrochloric acid method 1.

#### Sulphuric acid method 1 (Wahlen 1)

Direct transesterification was performed according to Wahlen et al. [19] with modifications: 2 ml screw-cap PP tube was filled with 30 ± 5 mg freeze dried biomass or vegetable oil, approx. 250 ± 30 mg (710–1180 µm diameter) acid-washed glass beads, and 600 µl of chloroform. 1.02 mg of C13:0 TAG internal standard in 100 µl of hexane was added to the PP tube. The fungal biomass was homogenized in a Percellys Evolution tissue homogenizer at 5500 rpm, 6 × 20 s cycles. The processed biomass was transferred into microwave glass reaction vessel by washing the PP tube with 2400 µl of chloroform (3 × 800 µl). The solvent in the microwave vessel was evaporated under nitrogen at 30 °C, and 2 ml of freshly prepared MeOH with 2% H_2_SO_4_ was added. A stir bar was added to the microwave-vessel, capped and microwaved at 80 °C for 20 min, with 10 s pre-stirring in an Initiator microwave synthesizer (Biotage AB, Sweden). After cooling to room temperature, 1 ml of saturated NaHCO_3_ solution, 1 ml of distilled water, and 0.88 ± 3 mg of C15:1 FAME internal standard in 100 µl of hexane were added to the reaction tube. The fatty acid methyl esters (FAMEs) were extracted by the addition of 2 ml hexane–chloroform (4:1 v/v) followed by 10 s vortex mixing. The reaction tube was centrifuged at 3000 rpm for 5 min at 4 °C, and the upper (organic) phase was collected in glass tubes. The hexane–chloroform extraction was performed thrice. The residual biomass was stored at − 20 °C for FTIR measurements. The organic phase in the glass tube was prepared for the GC measurement according to Hydrochloric acid method 1.

#### Sulphuric acid method 2 (Wahlen 2)

Direct transesterification was performed according to Sulphuric acid method 1, with one modification: The reaction was conducted at 80 °C for 60 min in a heating block, instead of at 80 °C for 20 min in a microwave oven.

#### Acetyl chloride method 1 (Lepage 1)

Direct transesterification was performed according to Lepage and Roy [38] with modifications: 2 ml screw-cap PP tube was filled with 30 ± 5 mg freeze dried biomass or vegetable oil, approx. 250 ± 30 mg (710–1180 µm diameter) acid-washed glass beads, and 600 µl of chloroform. 1.02 mg of C13:0 TAG internal standard in 100 µl of hexane was added to the PP tubes. The fungal biomass was homogenized in a Percellys Evolution tissue homogenizer at 5500 rpm, 6 × 20 s cycles. The processed biomass was transferred into a glass reaction tube by washing the PP tube with 2400 µl of chloroform (3 × 800 µl). The solvent in glass tube was evaporated under nitrogen at 30 °C, and 2 ml of freshly prepared acetyl chloride-methanol (5:100, v/v) was added. The reaction mixture was incubated at 90 °C for 1 h in a heating block, followed by cooling to room temperature. After cooling to room temperature, 2 ml of hexane, 1ml of distilled water, and 0.88 ± 3 mg of C15:1 FAME internal standard in 100 µl of hexane were added to the reaction tube. After 10 s vortex mixing, the reaction tube was centrifuged at 3000 rpm for 5 min at room temperature, and the organic phase was collected in a separate glass tube. The water phase was extracted two more times, but now by the addition of 2 ml hexane–chloroform mixture (4:1 v/v). The residual water phase (with residual biomass) was stored at − 20 °C for FTIR measurements. The organic phase in glass tubes was prepared for the GC measurement according to Hydrochloric acid method 1.

#### Acetyl chloride method 2 (Lepage 2)

Direct transesterification was performed according to Acetyl chloride method 1, with one modification: 2 ml of chloroform was added to the reaction mixture as co-solvent in addition to 2 ml of acetyl chloride-methanol.

#### Direct transesterification of fungal biomass from the microtiter plate cultivations

For the direct transesterification of fungal biomass from microtiter plate cultivations (*Mucor circinelloides*, *Umbelopsis vinacea*, *Absidia glauca*, *Lichtheimia corymbifera*, *Cunninghamella blakesleeana*, and *Amylomyces rouxii*) only Hydrochloric acid method 1 and Hydrochloric acid method 2 (with 90 min reaction time) were conducted. The Hydrochloric acid method 1 was implemented as stated above, while the Hydrochloric acid method 2 was slightly modified in respect to the internal standards. Specifically, 0.93 mg of C17:1 FAME internal standard in 100 µl of hexane were added to the GC vial (100 µl from a 9.3 mg/ml^− 1^ methyl 10(Z)-heptadecenoate; C_18_H_34_O_2_, C17:1 FAME, Larodan, Sweden). Each biological replicate was processed once with each of the two direct transesterification methods (i.e. two independent biological replicate measurements were obtained per method and per fungal strain).

### FTIR spectroscopy analysis

FTIR analyses of fungal biomass were performed before and after lipid extraction by both, the reflectance and transmittance infrared measurements. For measurement of the biomass after lipid extraction, biomass was washed and dried before FTIR measurement as described previously [14]. FTIR measurements were performed using a Vertex 70 FTIR spectrometer (Bruker Optik GmbH, Germany) equipped with a globar mid-IR source and a DTGS detector. The FTIR reflectance spectra were measured with a single reflectance-attenuated total-reflectance (SR-ATR) accessory High Temperature Golden gate ATR Mk II (Specac, United Kingdom). The ATR IR spectra were recorded with a total of 32 scans, spectral resolution of 4 cm^− 1^, and digital spacing of 1.928 cm^− 1^, over the range of 4000–600 cm^− 1^, using the horizontal SR-ATR diamond prism with 45° angle of incidence. Approximately 1 mg of sample was deposited onto the ATR crystal for each measurement, and each sample was measured in triplicates. Between each measurement a background (reference) spectrum was recorded using the sample-free setup. The FTIR transmittance spectra were measured using the High Throughput Screening eXTension (HTS-XT) unit (Bruker Optik GmbH, Germany) as described previously [28]. Fungal biomass was homogenized prior to the HTS FTIR measurements. Approximately 5 mg of biomass was transferred into 2 ml polypropylene tube containing 250 ± 30 mg of acid washed glass beads and 0.5 ml of distilled water, and homogenized by using Percellys Evolution tissue homogenizer (Bertin Technologies, France) with the following set-up: 5500 rpm, 6 × 20 s cycle. 10 µl of homogenized fungal biomass was pipetted onto an IR transparent 384-well silica microplate and dried at room temperature for 2 h. The HTS-FTIR spectra were recorded with a total of 64 scans, spectral resolution of 6 cm^− 1^, and digital spacing of 1.928 cm^− 1^, over the range of 4000–500 cm^− 1^, and an aperture of 5 mm. Spectra were recorded as the ratio of the sample spectrum to the spectrum of the empty IR transparent microplate. The OPUS software (Bruker Optik GmbH, Germany) was used for data acquisition and instrument control. ATR correction was performed by using *Extended ATR correction* algorithm of the OPUS software (see Additional file 1: Figure S3).

For chemical characterization of fungal biomass, a set of model compounds was measured by FTIR-ATR. Chitin, glyceryl trioleate (1,2,3-tri(cis-9-octadecenoyl)glycerol), and glucoronate (methyl 1,2,3,4-tetra-*O*-acetyl-β-d-glucuronate) were purchased from Merck-Sigma-Aldrich (Darmstadt, Germany) and used without further purification.

### GC‑FID total lipid content and fatty acid analysis

Determination of total lipid content (expressed as the wt% of total fatty acid methyl esters (FAMEs) of sample dry weight) and fatty acid composition (expressed as wt% of individual FAME of total FAMEs) were performed by using gas chromatography 7820A System (Agilent Technologies, USA), equipped with an Agilent J&W 121–2323 DB-23 column, 20m × 180 µm × 0.20 µm and a flame ionization detector (FID). Helium was used as a carrier gas. The total runtime for one sample was 36 min with the following oven temperature increase: initial temperature 70 °C for 2 min, after 8 min to 150 °C with no hold time, 230 °C in 16 min with 5 min hold time, and 245 °C in 1 min with 4 min hold time. The injector temperature was 250 °C and 1 µl of a sample was injected (30:1 split ratio, with split flow 30 ml/min). For the identification and quantification of fatty acids, the Supelco 37 Component FAME Mix (C4–C24 FAME mixture, Sigma-Aldrich, USA) was used as an external standard, in addition to C13:0 TAG and C15:1 FAME internal standards (see above, Direct transesterification of the fungal biomass). Measurements were controlled by the Agilent OpenLAB software (Agilent Technologies, USA).

### NMR spectroscopy analysis

Estimation of unreacted TAGs in the oil product after transesterification (expressed as the mole % of total FAMEs and TAGs) were performed by using nuclear magnetic resonance (NMR). The ^1^H-NMR spectra were recorded by an Ascend 400 spectrometer (Bruker BioSpin, Germany) at 400 MHz. Deuterated chloroform (CDCl_3_) was used as solvent for all the samples and the chloroform signal at 7.26 ppm was used as an internal standard. The conversion to FAMEs in the transesterification reaction was calculated based on the previously published methods [40, 41], by the methoxy protons of FAMEs at 3.65 ppm, and of the α-carbonyl methylene signals at 2.26 ppm.

The presence of unreacted TAGs was identified by the characteristic two doublet of doublets at 4.05–4.40 ppm from the methylene groups of the glycerol moiety of the triglyceride. Unreacted TAGs were quantified by the stoichiometric comparison of the integrals of the total α-carbonyl methylene signals of the total acyl lipids (FAMEs, free fatty acids and their derivatives, such as acylglycerols and glycerophospholipids, as well as minor components, such as fatty alcohols) and the signal of the glycerol moiety left after reaction. Error in the ^1^H-NMR measurement is estimated up to 5% [42]. TAGs were not estimated for Wahlen 1 method due to the overlap of many signals belonging to the microwave reaction products with the TAG-specific signals.

## Results and discussion

### Fungal strains

The studied species of filamentous fungi, namely *Mucor circinelloides*, *Umbelopsis vinacea*, *Absidia glauca*, *Lichtheimia corymbifera*, *Cunninghamella blakesleeana*, and *Amylomyces rouxii*, are considered as either model organisms (*Mucor circinelloides*) or oleaginous filamentous fungi of high industrial potential for production of microbial oils [43]. All species were grown under nitrogen-limitation to facilitate accumulation of lipids in the biomass. The assessment of the transesterification methods was conducted by using biomass of *M. circinelloides*, *U. vinacea*. These two Mucoromycota species are characterised by complex and resilient cell wall made of glucosamine and glucuronate biopolymers, and ability to accumulate extraordinarily high amount of lipids under the nitrogen-limited conditions. Moreover, they can store high amounts of intracellular polyphosphates in fungal cell wall and intracellular granules [28, 44]. The assessment of the optimised Lewis method was demonstrated on all six fungal strains.

### Direct transesterification methods and FAME yields

Research on transesterification is often focused on the base-catalysts, since they offer faster and milder reaction conditions compared to acid-catalysts [45]. However, base-catalysts have problems when dealing with a high content of free fatty acids and moisture in the sample. In addition to acylglycerols, biomass of oleaginous filamentous fungi and yeasts can contain high concentration of free fatty acids [14, 18, 46]. While transesterification of acylglycerols can be achieved with base-catalysts, esterification of free fatty acids cannot [47]. Since this can result in significant underestimate of total lipid yield in fungal biomass, transesterification methods for fungal biomass are almost exclusively acid-catalysed [18, 32–37].

Three common acid-catalysed direct transesterification methods were tested in this study: Lewis method (with hydrochloric acid catalyst) [10], Wahlen method (with sulphuric acid catalyst) [19], and Lepage method (with acetyl chloride catalyst) [38]. Out of the three methods, Lewis method is the simplest for handling since it requires relatively safe reagents that are tolerable to moisture (including wet biomass). Wahlen method has limited moisture tolerance and requires microwave heating which is a serious limitation for high-throughput analyses. Lepage method includes use of acetyl chloride, a highly flammable and corrosive substance, and the method requires a safe handling due to reactive nature of the acetyl chloride and water [19, 20, 38, 48]. In all three direct transesterification methods, hexane was used either as a co-solvent during the reaction or for extractive workup. Since hexane is a hazardous chemical, a safer alternatives, such as heptane, could be considered in future studies [49].

First, the three methods were used with minimal modifications, henceforth referred to as Lewis 1, Wahlen 1, and Lepage 1. Second, the three methods were extensively modified:

1. Lewis 2 had higher concentration of chloroform co-solvent: the standard method (Lewis 1) has solvent/co-solvent ratio 10:1, while the method 2 has this ratio 16:5. Moreover, non-polar solvent (chloroform) was added first, prior to bead beating preprocessing, followed by polar solvent (methanol) prior to the transesterification reaction. This modification was made to adjust the polarity of the extraction media for better solvation of the reacting lipids. Moreover, different reaction times were tested to optimise transesterification: 60, 90 and 120 min.
2. Wahlen 2 was conducted for 60 min in a heating block, instead for 20 min in a microwave oven as in the standard method (Wahlen 1). In both cases the reaction was conducted at 80 °C.
3. Lepage 2 was conducted with co-solvent (chloroform in 1:1 ratio to the main solvent), instead of conducting the reaction in pure acetyl chloride-methanol as in the standard method (Lepage 1).

**Fig. 1:**
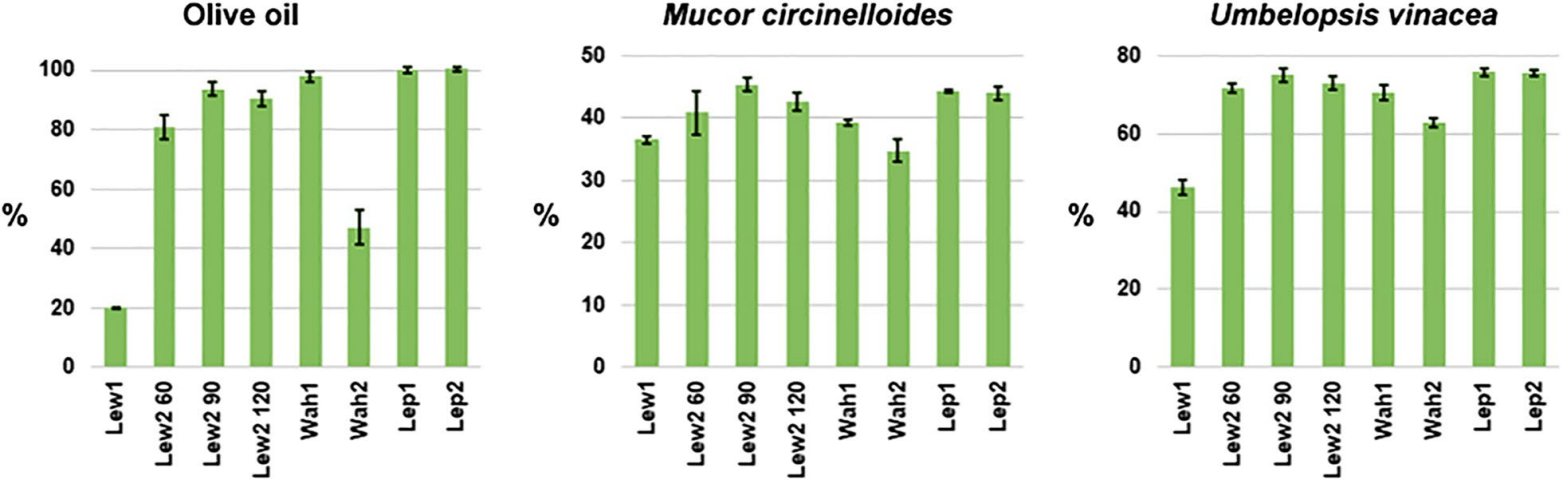
Total FAMEs yield estimate based on GC-FID measurements. The yield is calculated as percentage of dry biomass (%_w/w_), with one standard deviation error bars. Lew: Lewis method (with designated reaction times in minutes for method 2), Wah: Wahlen method, Lep: Lepage method

The gas chromatography (GC-FID) results show clear differences between the methods (Fig. 1, and Additional file 1: Table S1). For pure olive oil, the results show superiority of Wahlen 1 and both Lepage methods over Lewis methods. In particular, the difference is striking for Lewis 1, where lipid content was estimated to be only 20%, compared to 98% and 100% estimates for Wahlen 1 and Lepage 1 methods respectively. These results are consistent with the previously published studies that show superiority of Lepage method over Lewis method in lipid analysis of algal biomass [15, 16]. Modification of Lewis method (Lewis 2) resulted in greatly improved total lipid estimate, reaching 94% for 90 min reaction time (Fig. 1). However, modification of Wahlen method (Wahlen 2) resulted in halving of the lipid estimate (47%). Modification of Lepage method (Lepage 2) resulted in equally optimal lipid estimate of 100% as obtained by the standard method.

When transesterification was conducted on the fungal biomass,  differences between the methods were not so prominent as compared to the transesterification of pure oil (Fig. 1). Here, Lewis 2 and both Lepage methods were clearly superior to other methods. Regarding Lewis 2, the optimal reaction time was 90 min for all three types of samples (olive oil, and two types of fungal biomass). The optimal Lewis 2 (90 min), as well as Lepage methods, have estimated the same total lipid content in the dry biomass (i.e. total FAMEs) of approx. 45%_w/w_ for *Mucor circinelloides*, and 75%_w/w_ for *Umbelopsis vinacea*. Compared to these numbers, Wahlen 1 is underestimating the total FAME-converted lipids by 13% in case of *Mucor circinelloides* (39%_w/w_ total FAMEs), and 7% in case of *Umbelopsis vinacea* (70%_w/w_ total FAMEs). Lewis 1 is faring even worse, underestimating the total FAME-converted lipids by 18% in case of *Mucor circinelloides* (37%_w/w_ total FAMEs), and 39% in case of *Umbelopsis vinacea* (46%_w/w_ total FAMEs).

For the Lewis 1, the estimate of the total FAME-converted lipids was decreasing with the increase of oil content in the sample, dropping to only 20%_w/w_ in the case of pure vegetable oil (Fig. 1). The FAMEs yield has increased dramatically by modifying the solvent to co-solvent ratio. Thus, the proper amount of co-solvent is critical for optimal transesterification of samples by the Lewis method, and it can be concluded that the main problem with Lewis 1 was ineffective solvation. This is in agreement with the similar study on direct transesterification of microalgal biomass by a modified Lewis method [50]. It should be mentioned that the importance of solvation was noticed already in the original Lewis et al. study [10]. That study has shown that the order of solvent addition can influence the method yields, with better results obtained when the order was from non-polar to polar solvents, similar to our results.

An interesting result was observed with Wahlen 1, where the estimate of the total FAMEs-converted lipids was higher for the vegetable oil than for the fungal biomass. In particular, the total-FAMEs estimate for Wahlen 1 analysis of *Mucor circinelloides* biomass was just 39%_w/w_, compared to 44–45%_w/w_ obtained by Lepage and Lewis 2 methods (Fig. 1). It is known that *Mucor circinelloides* biomass has high content of polyphosphates [14]. These polyphosphates, as well as other cell wall biopolymers (glucosamines and glucuronates), probably compete with acylglycerols for acid-based catalyst during the reaction. Thus, higher concentration of catalyst is perhaps needed in the Wahlen method in order to compensate for this competing reaction. In the original study, Wahlen et al. have studied the influence of catalyst concentration on the transesterification, varying the amount of H_2_SO_4_ within the range 1.2–2.4%_v/v_ [19]. Although Wahlen et al. study showed that the concentration of the catalyst had a modest effect on the transesterification process, it should be noted that their study was conducted on very different type of biomass, specifically on green algae and diatoms than was the case in our study. Algae and diatoms in that study had significantly lower content of lipids (i.e. 7–27%_w/w_ relative to dry mass), than it was the case with *Mucor circinelloides* and *Umbelopsis vinacea* filamentous fungi (45 and 75% relative to dry mass, respectively). Modification of the Wahlen method (Wahlen 2), where the reaction was performed in a heating block instead of a microwave, showed that the microwave is a critical step for the efficient transesterification.

### Composition of extracted fungal lipids

**Fig. 2:**
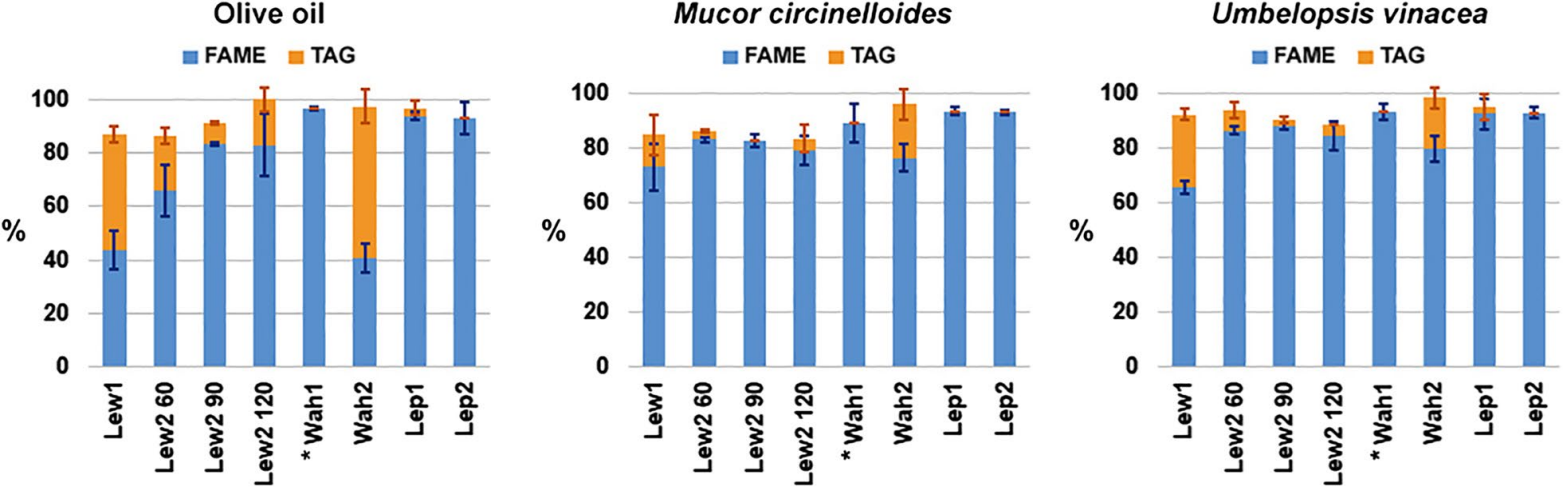
Total yield of FAME and TAG based on NMR analysis, calculated by specific signals in the extracted oil, with one standard deviation error bars. Yield is calculated as mol percentage (%_mol_) of total acyl lipids. Lew: Lewis method (with designated reaction times in minutes for method 2), Wah: Wahlen method, Lep: Lepage method. *TAGs were not estimated for Wahlen 1 method

In addition to the GC-FID analyses, we have conducted the NMR analyses of the extracted oils to verify the FAMEs yields, as well as to detect the residual triglycerides in the oils (Fig. 2). In general, the results are in agreement with the GC analyses. The small differences between the GC and NMR results can be ascribed to the inherent error of the NMR methodology [42], as well as to the difference in lipid estimate, reported as %_weight_ in the GC, and %_mol_ in the ^1^H-NMR. The NMR results confirm relatively low FAMEs yields for Lewis 1 and Wahlen 2 (Fig. 2). The NMR estimates of FAME and TAG for the analysis of *Mucor circinelloides* biomass by Lewis 1 are in agreement with the results from our previous study when lipid class composition was estimated by thin layer chromatography [14]. Furthermore, the NMR results show good transesterification yields for Wahlen 1, and both Lepage methods. Moreover, and as already indicated by the GC-FID results, modification of Lewis method resulted with greatly improved FAMEs yield and relatively small TAG residuals. In particular, TAG residuals were small when Lewis 2 method was conducted on the fungal biomass with 90 min reaction time.

Although the transesterification methods differed substantially regarding the total FAMEs yield, the fatty acid profiles of extracted fungal FAMEs were in agreement across the methods (Table 1). This shows that the transesterification conversion was proportional across all types of fatty acids, even for the methods with relatively low conversion rates, such as Lewis 1. For the pure vegetable oil, the differences in FAMEs profiles between the suboptimal methods (i.e. Lewis 1 and Wahlen 2) and the other methods were noticeable, but not extensive (approx. 3–12% relative difference in fatty acid compositions).

**Table 1:** Fatty acid profiles (%, normalized to total FAME), with one standard deviation error

FAME	Lew1	Lew2 60	Lew2 90	Lew2 120	Wah1	Wah2	Lep1	Lep2
**Olive oil**								
C16:0	11.58 ± 0.12	11.59 ± 0.01	11.18 ± 0.07	11.43 ± 0.12	11.19 ± 0.01	11.58 ± 0.03	11.19 ± 0.02	11.07 ± 0.00
C16:1	1.09 ± 0.04	0.91 ± 0.01	0.90 ± 0.01	0.88 ± 0.01	0.87 ± 0.00	1.01 ± 0.01	0.88 ± 0.01	0.86 ± 0.00
C18:0 + C18:1n9c	76.39 ± 0.15	78.48 ± 0.17	78.54 ± 0.31	78.31 ± 0.98	79.58 ± 0.08	78.05 ± 0.06	79.50 ± 0.08	79.28 ± 0.02
C18:2n6c	7.26 ± 0.12	6.58 ± 0.04	6.64 ± 0.05	6.47 ± 0.08	6.38 ± 0.01	7.05 ± 0.05	6.43 ± 0.04	6.81 ± 0.01
C18:3n3	0.83 ± 0.03	0.68 ± 0.01	0.70 ± 0.01	0.66 ± 0.00	0.66 ± 0.00	0.78 ± 0.01	0.67 ± 0.00	0.65 ± 0.00
***M. circinelloides***								
C14:0	1.54 ± 0.01	1.48 ± 0.14	1.48 ± 0.26	1.49 ± 0.00	1.51 ± 0.03	1.63 ± 0.00	1.49 ± 0.04	1.49 ± 0.05
C16:0	17.33 ± 0.06	17.18 ± 0.12	17.17 ± 0.03	17.25 ± 0.03	17.32 ± 0.00	17.33 ± 0.04	17.35 ± 0.04	17.41 ± 0.15
C16:1	4.35 ± 0.01	4.18 ± 0.05	4.23 ± 0.03	4.19 ± 0.06	4.34 ± 0.02	4.54 ± 0.03	4.29 ± 0.02	4.28 ± 0.03
C17:0	0.81 ± 0.01	0.84 ± 0.01	0.82 ± 0.00	0.84 ± 0.01	0.85 ± 0.01	0.80 ± 0.01	0.82 ± 0.00	0.83 ± 0.01
C17:1	0.71 ± 0.00	0.70 ± 0.01	0.71 ± 0.00	0.71 ± 0.01	0.69 ± 0.00	0.72 ± 0.00	0.71 ± 0.01	0.71 ± 0.01
C18:0 + C18:1n9c	50.14 ± 0.15	50.20 ± 0.30	50.28 ± 0.09	50.41 ± 0.22	50.56 ± 0.03	49.44 ± 0.19	50.85 ± 0.14	51.05 ± 0.48
C18:2n6t	0.69 ± 0.02	0.65 ± 0.01	0.69 ± 0.00	0.66 ± 0.01	0.65 ± 0.00	0.67 ± 0.00	0.65 ± 0.00	0.65 ± 0.01
C18:2n6c	12.51 ± 0.02	12.28 ± 0.07	12.36 ± 0.04	12.31 ± 0.05	12.36 ± 0.01	12.74 ± 0.02	12.38 ± 0.04	12.73 ± 0.10
C18:3n6	9.92 ± 0.02	9.54 ± 0.05	9.62 ± 0.03	9.56 ± 0.01	9.71 ± 0.04	10.38 ± 0.07	9.69 ± 0.06	9.65 ± 0.07
***U. vinacea***								
C14:0	0.71 ± 0.00	0.65 ± 0.00	0.64 ± 0.01	0.64 ± 0.00	0.64 ± 0.03	0.69 ± 0.00	0.63 ± 0.01	0.64 ± 0.02
C16:0	27.85 ± 0.10	27.49 ± 0.01	27.34 ± 0.03	27.40 ± 0.03	27.52 ± 0.01	27.76 ± 0.06	27.60 ± 0.03	27.41 ± 0.07
C16:1	3.09 ± 0.02	2.92 ± 0.00	2.88 ± 0.00	2.90 ± 0.01	2.93 ± 0.02	3.06 ± 0.01	2.92 ± 0.01	2.87 ± 0.01
C18:0 + C18:1n9c	57.11 ± 0.11	58.13 ± 0.04	57.96 ± 0.05	58.13 ± 0.12	58.33 ± 0.06	57.88 ± 0.07	58.50 ± 0.14	58.24 ± 0.15
C18:2n6c	5.17 ± 0.01	4.93 ± 0.02	4.92 ± 0.03	4.94 ± 0.01	4.84 ± 0.03	5.01 ± 0.03	4.83 ± 0.02	5.17 ± 0.01
C18:3n6	3.60 ± 0.01	3.29 ± 0.01	3.30 ± 0.01	3.28 ± 0.02	3.27 ± 0.02	3.49 ± 0.03	3.28 ± 0.01	3.25 ± 0.01
C20:0	0.62 ± 0.00	0.72 ± 0.00	0.72 ± 0.00	0.74 ± 0.00	0.72 ± 0.01	0.65 ± 0.00	0.72 ± 0.01	0.73 ± 0.00

Lew: Lewis method (with designated reaction times in minutes for method 2), Wah: Wahlen method, Lep: Lepage method

### Assessment of the residual lipids in the biomass after the transesterification

**Fig. 3:**
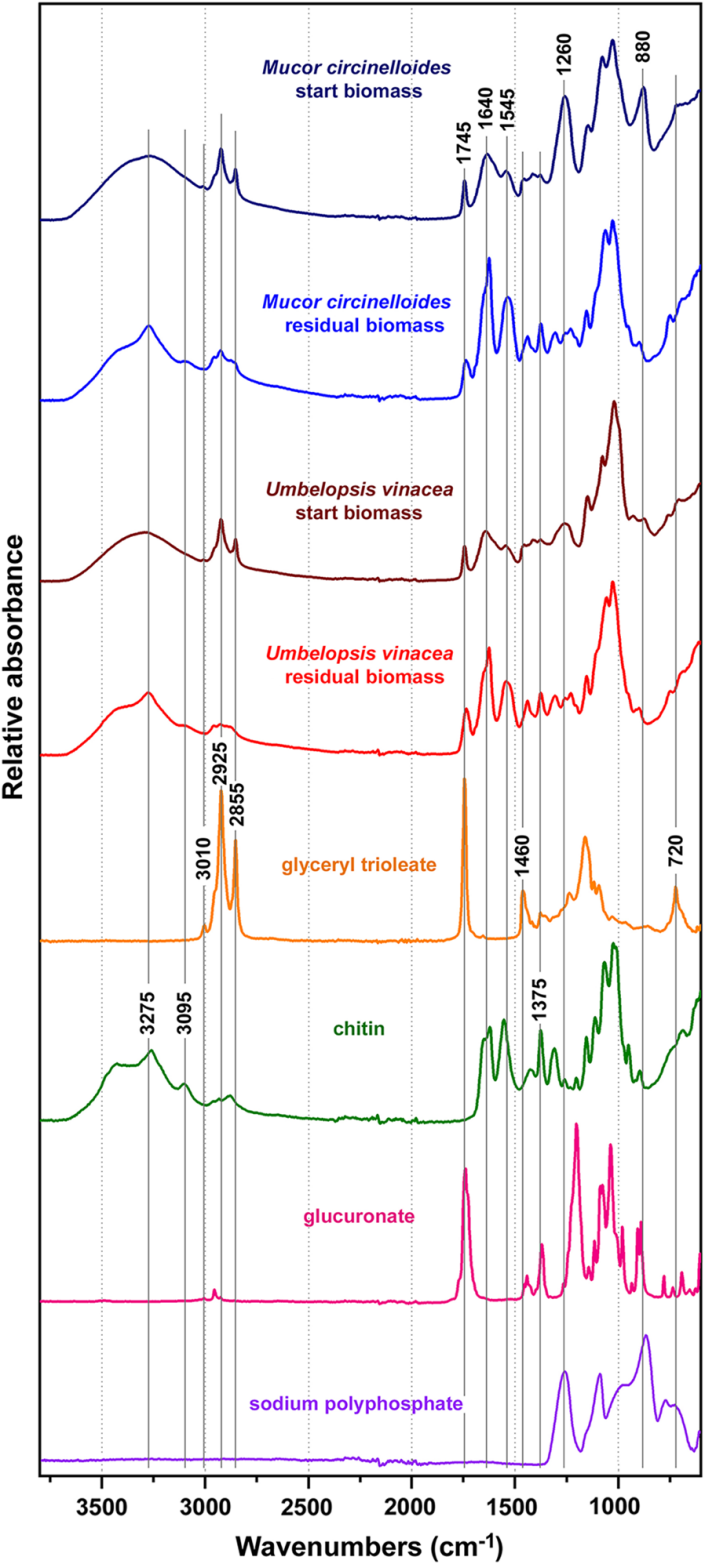
FTIR spectra of fungal biomass before and after direct transesterification (Lewis 2 method with 90 min reaction time), and of model compounds (glyceryl trioleate, chitin, glucuronate, and sodium polyphosphate). Spectra are plotted with an offset for better viewing

Assessment of the residual lipids in the fungal biomass after the transesterification reaction was done by FTIR spectroscopy, as demonstrated recently [14]. Following the transesterification reaction, the reaction mixture was processed by the follow-up extractive workup, specifically organic-water biphasic extraction. For all direct transesterification methods, the residual fungal biomass was isolated from the water phase by centrifugation, washed three times with distilled water, and measured by the FTIR spectroscopy (Fig. 3 and Additional file 1: Figure S1). The lipid content of the intact fungal biomass can be estimated based on the signals associated with triglycerides: C–H stretching vibrations (=C–H stretching at 3010 cm^−1^; C–H stretching in –CH_3_ and –CH_2_ at 2954, 2925 and 2855 cm^−1^), C=O stretching in esters (1745 cm^−1^), CH_2_ bending (1460 cm^−1^), C–O–C stretching in esters (1200–1070 cm^−1^) and CH_2_ rocking (720 cm^−1^) [14, 23]. In addition to the lipid-related signals, the biomass before extraction shows signals at 1640 and 1545 cm^−1^ related to proteins (C=O stretching in amides (amide I), and C–N–H vibration (amide II) respectively). Finally, signals at 1260 and 880 cm^−1^, related to polyphosphate P–O and P=O stretching [51], could be explained by the accumulation of polyphosphates in Mucoromycota fungi [28, 52, 53]. The FTIR spectra indicate that biomass of *Mucor circinelloides* has higher content of polyphosphates than *Umbelopsis vinacea* (Fig. 3, Additional file 1: Figure S2).

The infrared spectra of residual biomass (biomass after extraction) show that the majority of lipids, in particular triglycerides, were extracted for all direct transesterification methods (Fig. 3). The predominant spectral features are signals associated with cell wall carbohydrates, namely chitin and chitosan (N–H stretching at 3275 and 3095 cm^−1^, C=O stretching in amides at 1660 and 1625 cm^−1^ (amide I), and C–N–H vibration at 1524 cm^−1^ (amide II), CH and CH_3_ bending at 1375 cm^−1^, C–O and C–O–C stretching at 1200–1000 cm^− 1^, CH_3_ bending at 950 cm^−1^), glucans (C–O and C–O–C stretching at 1200–1000 cm^−1^) and glucuronans (C=O stretching in esters at 1735 cm^−1^, C–O and C–O–C stretching at 1200–1000 cm^−1^) [54–56]. The spectra of biomass after extraction are clearly devoid of signals associated with triglycerides and polyphosphates. It can be presumed that acidic conditions of all tested direct transesterification methods have led to hydrolysis of cell-wall polyphosphates. Thus, acid catalyst is not only important for the transesterification reaction, but it also facilitate degradation of cell wall, leading to efficient extraction of lipids. This is in agreement with our recent study that showed satisfactory degradation of fungal cell wall by bead beating and acid pretreatment [14].

However, the presence of high concentration of polyphosphates in fungal biomass, in particular in *Mucor circinelloides*, probably hinders the transesterification process of TAGs due to the competing acid-based hydrolysis of polyphosphates. All the direct transesterification methods are conducted in relatively polar solvent, which facilitates extraction of phosphate compounds. It has been reported that phosphates, in the form of phospholipids and polyphosphates, hinder transesterification of acylglycerols [57]. This can result in lower FAME yields, as already commented for Wahlen 1 method.

In this study we have used both reflectance (ATR) and transmittance (HTS) FTIR methods for obtaining the IR spectra. Considering that the two methods result with qualitatively different spectra (see Additional file 1: Figure S2), it is important to clarify why these differences occur. The main difference between the ATR and HTS infrared spectra is the difference in intensity of the absorption bands. The high-wavenumber bands have significantly lower intensity when measured with the ATR method than with the HTS method. The reason for this effect is wavelength-dependence of the IR-beam penetration depth when measuring with the ATR method. The penetration depth (i.e. IR-beam pathlength through the sample) is higher for higher wavelengths, thus the spectra show higher intensity of the low-wavenumber absorbance bands. The effect of the wavelength dependency of the penetration depth is routinely corrected with spectral acquisition software (in our case with Bruker OPUS software) when one wants to compare spectra measured using reflectance (ATR-FTIR) and transmittance (HTS-FTIR) techniques. Thusly corrected ATR spectrum of *Mucor circinelloides* intact biomass is shown in Additional file 1: Figure S3. However, such correction is valid only for homogenous samples, for example *Mucor circinelloides* residual biomass (due to homogenization step with bead beating). In case of intact fungal biomass, chemical components have uneven spatial allocation. For example, cell wall of *Mucor circinelloides* is predominantly made of glucosamine polysaccharides (chitin and chitosan), while cell interior is dominated by lipids [28]. As a result, ATR spectrum will overrepresent chemical components present in the surface area of the sample, such as chitin, and underestimate lipids and other chemical components present in the interior of fungal hyphae. Since HTS transmittance method requires sample homogenization, the resulting spectra show less bias towards different chemical components present in the sample. In general, it is easier to notice spectral differences related to lipids when using HTS method [14], though both FTIR methods are equally useful for assessment of residual lipids in fungal biomass after transesterification reaction.

### Importance of the internal standard

**Fig. 4:**
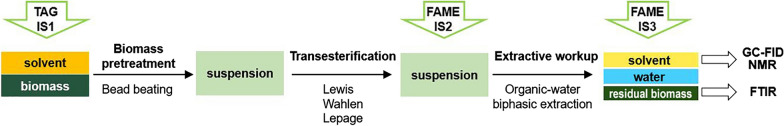
Schematic overview of the study design, with direct transesterification steps and methods, and internal standards (IS1–C13:0 TAG; IS2–C15:1 FAME; IS3–C17:1 FAME)

All studied transesterification methods have three crucial steps: (1) biomass pretreatment, (2) transesterification reaction, and (3) extractive workup (Fig. 4). In this study, all three main steps where controlled by the internal standards for the GC-FID analysis. All three internal standards comprised fatty acids (C13:0, C15:1 and C17:1) that are either not present in fungal oils or present as minor components.

Internal standard is crucial for estimating the total FAME-converted lipids. Namely, total lipid yield is often being estimated as total FAMEs yield by GC-FID [10, 15, 16, 20, 21, 32, 50]. As stated previously, this is valid only if the predominant lipid classes present in the biomass can be converted into FAMEs (for example, fatty acids, acylglycerols and glycerophospholipids). In general, oleaginous microorganisms contain mainly such type of lipids, in particular triacylglycerols. The total lipid yield can be also estimated gravimetrically [18, 34–36, 44]. However, gravimetric lipid quantification is inherently variable and inaccurate due to the extraction of non-lipid compounds, such as proteins, and thus can over- or underestimate the lipid content [58].

For the accurate assessment of FAMEs from fungal biomass it is important that the appropriate internal standard (i.e. the internal standard of similar chemical composition to the predominant analyte lipids) is added prior to the transesterification reaction [16]. In our study, the main internal standard was C13:0 TAG (glyceryl tritridecanoate), since the predominant class of lipids in oleaginous filamentous fungi are triacylglycerols [14, 59], and it was added at the very beginning of all transesterification methods, before the biomass pretreatment (Fig. 4). Biomass pretreatment was done by bead beating in order to accomplish cell wall disruption, biomass grinding and homogenization. Moreover, the pretreatment also served to provide good homogenization of the biomass (or vegetable oil sample) with the C13:0 TAG internal standard.

The second internal standard was C15:1 FAME (methyl 10(Z)-pentadecenoate), which was added after the transesterification reaction in order to assess the transesterification yield into FAMEs (Fig. 4). To be precise, by comparing the actual value of the added C15:1 FAME with the calculated estimate of the C15:1 FAME based on the C13:0 TAG internal standard, we were able to estimate the conversion of C13:0 TAG into C13:0 FAME (Table 2). The results show that both Lepage methods were able to convert almost all C13:0 TAG into C13:0 FAME, as previously indicated by the NMR analysis (Table 2; Fig. 2). Lewis methods had internal standard conversion into FAME of approx. 90%, while Wahlen methods had between 79 and 96%, depending on the type of sample (Table 2). Interestingly, Wahlen 1 had much lower conversion yield in the presence of fungal biomass than what was the case for the vegetable oil. This is yet another indication that polyphosphates and cell wall polysaccharides are probably competing with acylglycerols for acid-based catalyst, thus hindering the transesterification.

**Table 2:** GC-FID estimate of conversion of C13:0 TAG internal standard into C13:0 FAME, expressed as percentage with one standard deviation error. (based on the C15:1 FAME internal standard)

	Lew1	Lew2 60	Lew2 90	Lew2 120	Wah1	Wah2	Lep1	Lep2
Olive oil	89 ± 4	88 ± 3	91 ± 1	88 ± 3	96 ± 2	79 ± 10	96 ± 1	97 ± 0
*M. circinelloides*	94 ± 2	92 ± 2	92 ± 0	92 ± 2	84 ± 1	91 ± 2	97 ± 1	96 ± 0
*U. vinacea*	91 ± 2	91 ± 2	92 ± 1	89 ± 1	84 ± 3	90 ± 2	96 ± 1	97 ± 1

Importantly, high conversion yield of the internal standard C13:0 TAG into C13:0 FAME was accomplished even for rather ineffective direct transesterification methods, such as Lewis 1 and Wahlen 2 methods. For example, for these two methods the conversion yield of the internal standard into FAME was approx. 80–90%, while the conversion of vegetable oil TAGs into FAMEs was only 20–40%. In general, incomplete transesterification is not detrimental for estimating total FAME content in the biomass since the error created by incomplete transesterification can be corrected by the use of the internal standard [60]. However, this is only valid as long as both internal standard and the biomass lipids have the same conversion yield into FAMEs, which was not the case here. Therefore, our finding is of great importance for the lipid research studies based on the lipid yield determination by GC analysis since it demonstrates that extremely inaccurate total FAME content estimates are obtained when an internal standard and lipid analytes have different conversion yields into FAMEs. For example, for the Lewis 1, the total FAMEs content was underestimated by 20–40% in fungal biomass, and by 80% (i.e. five times lower) in vegetable oil.

It can be presumed that the reason for the difference in conversion yield between the vegetable oil TAGs and the internal standard C13:0 TAG is due to the different solvation of these triacylglycerols. Specifically, the internal standard was already well solvated in hexane when it was added to the sample, while solvation of sample lipids commenced during the pretreatment step. In the lipid research studies, an internal standard is often being added completely solvated in a solution. In most cases, such internal standard solution is based on the reaction solvent [15, 21, 38]. Alternatively, internal standard solution is based on a third type of solvent, different than in the main solvent/co-solvent system [16, 32]. The reason for this is ease of handling and time-saving. However, as indicated by our results, this can lead to incorrect estimate of total FAMEs content in the biomass. It is worth noting that a number of studies use internal standard in the form of FAME [10, 50], instead of TAG or free fatty acid, which can result in underestimate of the total FAME content in the biomass if analyte lipids are not completely converted into FAMEs.

Lewis 2 method clearly demonstrates that addition of co-solvent at the very beginning of the pretreatment phase enables good solvation and extraction of lipids from the biomass during the bead beating cell disruption and subsequent reaction. Moreover, it enables good homogenization of cellular (and vegetable) lipids with the internal standard. Thus, even though Lewis 2 has somewhat lower conversion yield of TAGs to FAMEs, compared to the Lepage methods, it offers precise estimate of lipids in the biomass since the internal standard and the biomass lipids have the same conversion yield. However, this only demonstrates that Lewis 2 is reliable in the lipid research, where total lipid yields (i.e. FAME content) and fatty acid compositions are of main importance. Regarding industrial production of biodiesel, Lewis 2 is not as suitable as Lepage method due to lower conversion yield of TAGs into FAMEs, as indicated by the NMR analysis (Fig. 2).

The third internal standard used in the study was C17:1 FAME (methyl 10(Z)-heptadecenoate), which was added directly before the GC measurements to assess the lipid losses during the extractive workup (Fig. 4 and Additional file 1: Table S3). The extractive workup includes water-phase treatment of the reaction media following the transesterification reaction in order to facilitate separation of the hydrophobic lipids from the hydrophilic compounds. This internal standard control was conducted only for the transesterification of fungal biomass from the microtiter plate cultivations. Comparison of the actual values of the added C15:1 and C17:1 FAMEs showed that there was no noteworthy loss of lipids during the workup (Additional file 1: Table S2). For *Mucor circinelloides* and *Umbelopsis vinacea*, the minor difference between the measured FAME internal standards can be attributed to small intrinsic amount of C17:1 in the fungal biomass, which was detected by analysing the biomass with Lewis 1 without C17:1 internal standard (Additional file 1: Table S3).

### Modified Lewis method and screening of oleaginous filamentous fungi

Our previous study has identified several promising filamentous fungal strains for the production of high-value PUFA and biodiesel [26]. However, these results were obtained by applying Lewis 1 as described here. The assessment of fungal biomass after transesterification reaction by FTIR indicates complete extraction of triglyceride lipids and hydrolysis of cell-wall polyphosphates (Additional file 1: Figure S4). Implementation of Lewis 2 on the selected fungal strains cultivated in the high-throughput Duetz-MTP screening system, indicates that the previous study has underestimated total FAME yields by a large extent (Table 3). Specifically, the estimates for the lipid yields obtained by Lewis 2 are 34–84% higher than the values obtained by Lewis 1. The difference in total FAMEs yield had no impact on the fatty acid profiles of extracted fungal FAMEs since for both Lewis methods the FAME profiles were in large agreement (Additional file 1: Table S3). This was expected considering our other results (Table 1).

**Table 3:** Lipid yield (%) from GC-FID for two different biological replicates per strain

Sample/method	Biological replicate 1		Biological replicate 2	
	Lew1	Lew2 90	Lew1	Lew2 90
*Mucor circinelloides*	38.25	48.31	29.73	44.45
*Umbelopsis vinacea*	37.57	64.02	33.99	65.22
*Cunninghamella blakesleeana*	23.42	42.90	22.67	42.00
*Lichtheimia corymbifera*	24.29	37.59	33.07	37.18
*Amylomyces rouxii*	23.50	36.61	27.62	36.43
*Absidia glauca*	44.40	56.33	39.23	59.86

Lew: Lewis 1 and 2 methods (with designated reaction time in minutes for method 2)

## Conclusions

The study has shown that standard Lepage method (Lepage 1) and the optimised Lewis method (Lewis 2 at 90 °C) are suitable for lipid analysis of oleaginous filamentous fungi. Comparing the two methods, the optimised Lewis method uses reagents which are easier to prepare and are much less water-sensitive than the reagent (acetyl chloride) in Lepage method. Water sensitivity of acetyl chloride requires drying of solvents as well as thorough freeze-drying of biomass. Wahlen method shows certain deficiencies when dealing with the fungal biomass, indicating a significant matrix effect probably caused by the presence of polyphosphates and polysaccharides in the fungal cells. The significant difference in lipid yields results, obtained by optimised and standard Lewis methods, indicates that some of the previously reported lipid yields must be corrected upwards. This could have important biotechnological implications for production of high-value (PUFA-rich) oils, as well as biodiesel, since it would indicate that some fermentation processes are more economically viable than previously estimated. Finally, the study demonstrates value of biomass monitoring by FTIR, importance of optimal solvent to co-solvent ratio, as well as careful selection and implementation of internal standards for gas chromatography.

## Supplementary Information

**Additional file 1: Table S1.** Lipid yield from GC-FID. **Figure S1.** FTIR spectra of fungal biomass before and after transesterification reactions. **Figure S2.** FTIR spectra of *Mucor circinelloides* and *Umbelopsis vinacea* fungal biomass before and after transesterification reaction. **Figure S3.** FTIR ATR spectra with ATR correction for IR-beam penetration depth. **Table S2.** Ratio of normalised measured FAME internal standards. **Figure S4.** FTIR HTS spectra of fungal biomass after transesterification reaction. **Table S3.** Fatty acid profiles for Lewis 1 and the optimal Lewis 2 methods.

## Data Availability

The data generated for this study are available in Additional file 1.
