# Supplementary material for: Evaluation and optimisation of direct transesterification methods for the assessment of lipid accumulation in oleaginous filamentous fungi
Source: Microb Cell Fact. 2021 Mar 3;20:59. doi: 10.1186/s12934-021-01542-1 (PMC7931520; doi:10.1186/s12934-021-01542-1)
Supplement: Supplementary file 1 — Additional file 1: Table S1. Lipid yield from GC-FID. Figure S1. FTIR spectra of fungal biomass before and after transesterification reactions. Figure S2. FTIR spectra of Mucor circinelloides and Umbelopsis vinacea fungal biomass before and after transesterification reaction. Figure S3. FTIR ATR spectra with ATR correction for IR-beam penetration depth. Table S2. Ratio of normalised measured FAME internal standards. Figure S4. FTIR HTS spectra of fungal biomass after transesterification reaction. Table S3. Fatty acid profiles for Lewis 1 and the optimal Lewis 2 methods. [file 12934_2021_1542_MOESM1_ESM.pdf]

Supplementary Material

Evaluation and optimisation of direct transesterification methods for the assessment of lipid accumulation in oleaginous filamentous fungi

Anne Marie Langseter[1] (anne.langseter@nmbu.no), Simona Dzurendova[1] (simona.dzurendova@nmbu.no), Volha Shapaval[1] (volha.shapaval@nmbu.no), Achim Kohler[1] (achim.kohler@nmbu.no), Dag Ekeberg[2] (dag.ekeberg@nmbu.no), Boris Zimmermann[1]* (boris.zimmermann@nmbu.no).

[1]Faculty of Science and Technology, Norwegian University of Life Sciences, Postbox 5003, 1432 Ås, Norway
[2]Faculty of Chemistry, Biotechnology and Food Science, Norwegian University of Life Sciences, P.O. Box 5003, 1432 Ås, Norway
Correspondence address: Faculty of Science and Technology, Norwegian University of Life Sciences, Postbox 5003, 1432 Ås, Norway

*Corresponding author:
Boris Zimmermann
Faculty of Science and Technology
Norwegian University of Life Sciences
Drøbakveien 31, 1432 Ås, Norway.
Tel: +47 6723 1576
Fax: +47 6496 5001
E-mail: boris.zimmermann@nmbu.no

Table of Contents **Page**

Table S1. Lipid yield from GC-FID S-2
Figure S1. FTIR spectra of fungal biomass S-3
Figure S2. FTIR spectra of fungal biomass: *M. circinelloides* and *U. vinacea* S-4
Figure S3. FTIR ATR: correction for IR-beam penetration depth S-5
Table S2. Ratio of normalised measured FAME internal standards S-6
Figure S4. FTIR HTS spectra of fungal biomass for six fungal strains S-7
Table S3. Fatty acid profiles for six fungal strains S-8

Table S1. Lipid yield from GC-FID (with standard deviation values)

	Lew1	Lew2 60	Lew2 90	Lew2 120	Wah1	Wah2	Lep1	Lep2
Olive oil	20.08 ±0.26	80.75 ±4.18	93.62 ±2.14	90.37 ±2.51	97.96 ±1.73	47.02 ±5.76	100.11 ±1.18	100.44 ±0.80
M. circinelloides	36.54 ±0.61	40.78 ±3.59	45.24 ±1.09	42.29 ±1.46	39.24 ±0.52	34.72 ±1.79	44.32 ±0.30	43.92 ±1.09
U. vinacea	46.20 ±1.80	71.65 ±1.21	74.90 ±1.60	72.90 ±1.81	70.44 ±1.83	62.58 ±1.14	75.66 ±0.88	75.54 ±0.78

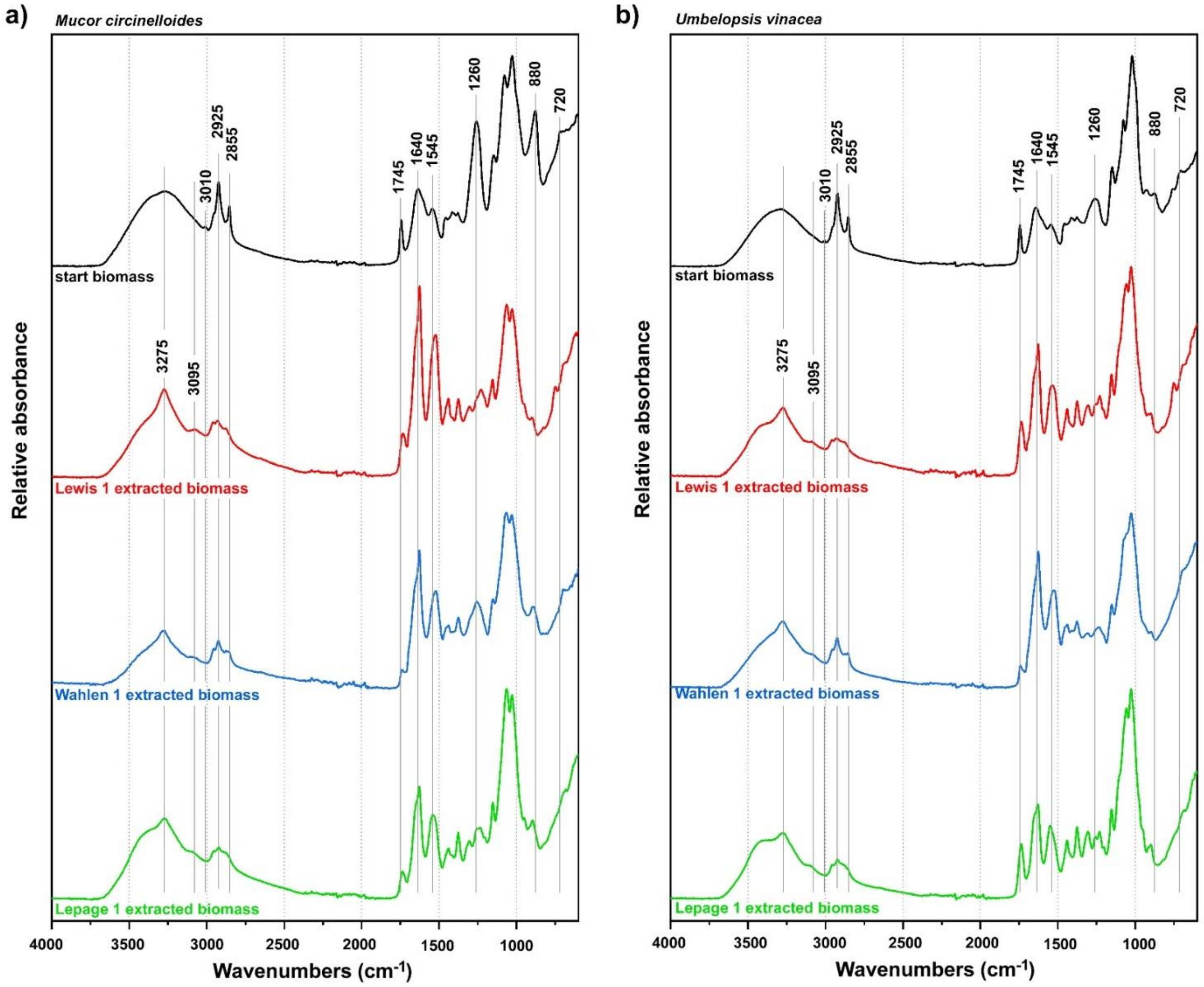

Figure S1. FTIR spectra of fungal biomass before and after transesterification reactions (Lewis 1, Wahlen 1, and Lepage 1 methods): a) *Mucor circinelloides*, b) *Umbelopsis vinacea*.

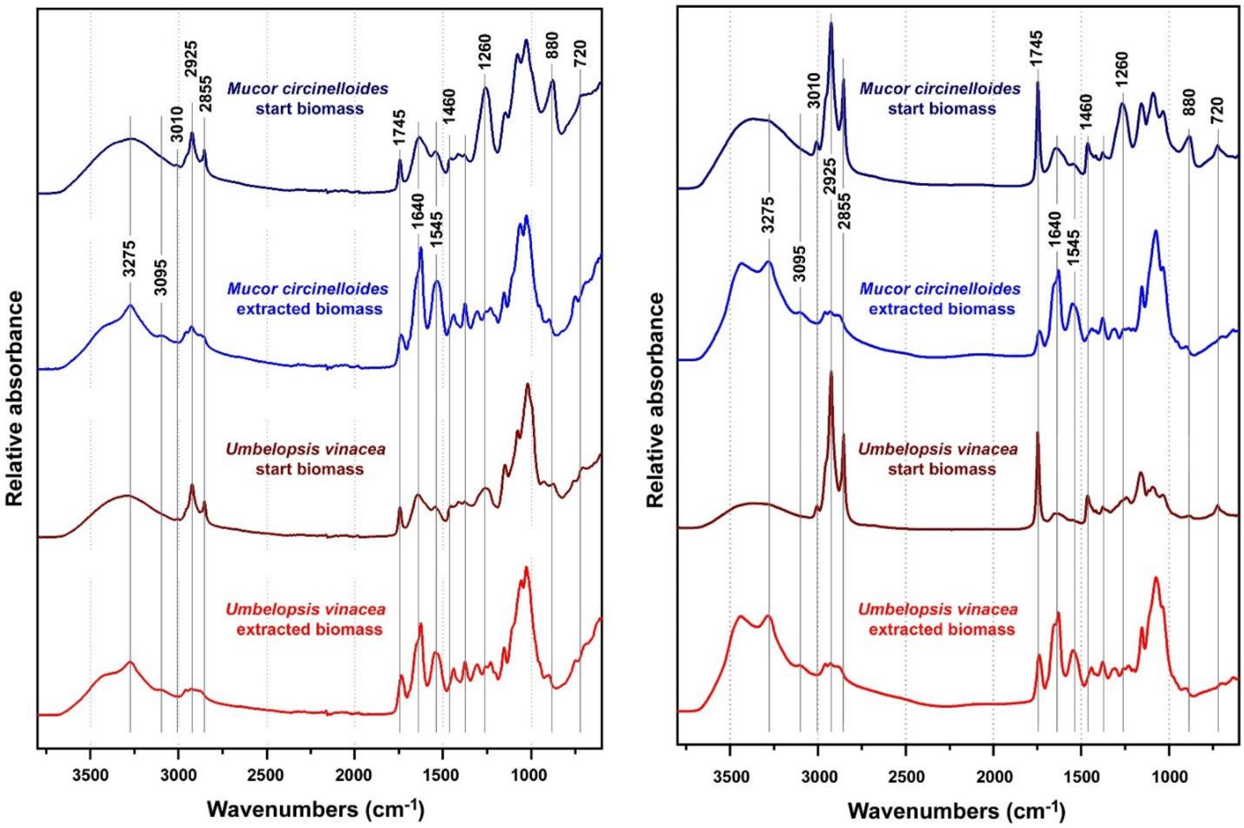

Figure S2. ATR (left) and HTS (right) FTIR spectra of *Mucor circinelloides* and *Umbelopsis vinacea* fungal biomass before and after transesterification reaction (Lewis 2 method with 90 min reaction time).

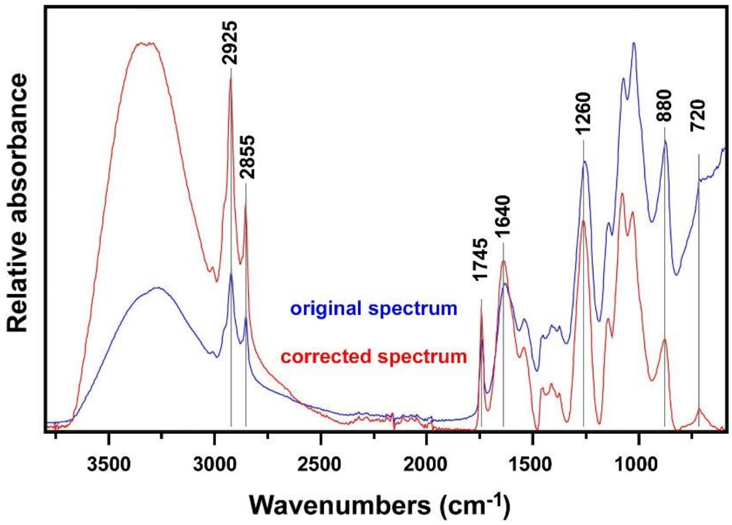

Figure S3. FTIR ATR spectra of *Mucor circinelloides* fungal biomass before transesterification reaction: original measured spectrum (blue) and after ATR correction for IR-beam penetration depth.

Table S2. The ratio of normalised measured FAME internal standards (C15:1 and C17:1) for Lewis 1 and optimal Lewis 2 methods (Lewis 2 method, 90 min reaction time). Normalised values were obtained by dividing the GC-FID measured values (based on C13:0 TAG internal standard) with the actual values of added FAME internal standards.

	Biological replicate 1	
**Sample \ Method**	**Lew1**	**Lew2 90**
Mucor circinelloides	0.94	0.93
Umbelopsis vinacea	0.95	0.96
Cunninghamella blakesleeana	0.95	0.96
Lichtheimia corymbifera	1.00	0.99
Amylomyces rouxii	0.98	0.96
Absidia glauca	0.97	0.99

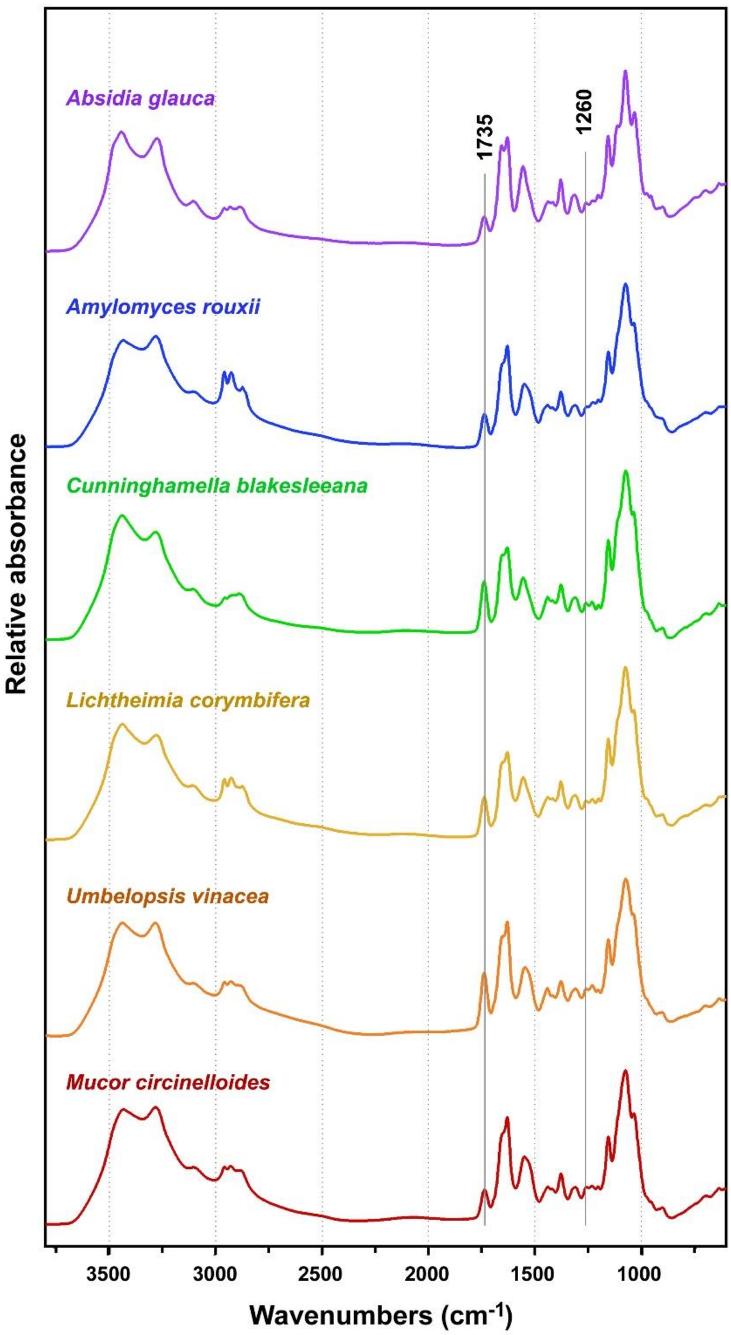

Figure S4. HTS FTIR spectra of *Absidia glauca*, *Amylomyces rouxii*, *Cunninghamella blakesleeana*, *Lichtheimia corymbifera*, *Umbelopsis vinacea*, and *Mucor circinelloides* fungal biomass after transesterification reaction (Lewis 2 method with 90 min reaction time).

Table S3a Fatty acid profiles for Lewis 1 and the optimal Lewis 2 methods (90 min reaction time for method 2). IS designates internal standard.

	Biological replicate 1		Biological replicate 2	
FAME	Lew1	Lew2 90	Lew1	Lew2 90
M. circinelloides				
C14:0	2.14	1.79	1.62	1.44
C16:0	17.95	17.76	17.53	17.48
C16:1	3.78	3.76	3.59	3.42
C17:0	0.97	0.97	0.73	0.76
C17:1	0.56	IS	0.50	IS
C18:0 + C18:1n9c	47.96	48.96	47.82	50.13
C18:2n6t	0.56	0.60	0.47	0.47
C18:2n6c	12.77	13.29	13.68	13.36
C18:3n6	10.06	10.13	11.33	10.47
U. vinacea				
C14:0	0.67	0.59	0.70	0.59
C16:0	24.87	25.10	23.92	24.25
C16:1	2.07	1.98	2.03	1.87
C17:1	0.23	IS	0.25	IS
C18:0 + C18:1n9c	57.71	59.81	55.89	60.46
C18:2n6c	6.32	6.00	6.58	5.91
C18:3n6	4.28	3.88	4.80	3.97
C20:0	0.79	0.89	0.74	0.91
C. blakesleeana				
C14:0	0.69	0.63	0.53	0.64
C16:0	16.51	15.44	17.66	18.56
C16:1	0.56	0.58	0.59	0.58
C18:0 + C18:1n9c	53.59	54.32	52.18	54.86
C18:2n6c	14.36	13.45	14.38	12.84
C18:3n6	9.36	8.81	9.83	7.74
C20:1n9	0.67	0.68	0.65	0.57
C22:0	0.51	0.65	0.53	0.62
C24:0	1.79	2.29	1.75	1.89

Table S3b Fatty acid profiles for Lewis 1 and the optimal Lewis 2 methods (90 min reaction time for method 2). IS designates internal standard.

	Biological replicate 1		Biological replicate 2	
FAME	Lew1	Lew2 90	Lew1	Lew2 90
L. corymbifera				
C14:0	0.56	0.49	0.52	0.54
C16:0	24.60	24.00	24.51	24.96
C16:1	0.99	0.98	0.92	0.97
C18:0 + C18:1n9c	55.31	56.94	57.02	57.07
C18:2n6c	11.05	10.57	10.44	10.45
C18:3n6	4.38	4.02	3.90	3.81
C20:0	0.46	0.54	0.55	0.51
A. rouxii				
C14:0	1.83	1.39	1.55	1.39
C16:0	21.32	21.54	21.53	21.48
C16:1	2.27	1.88	1.99	1.90
C17:1	0.38	IS	0.27	IS
C18:0 + C18:1n9c	45.57	46.55	45.48	46.66
C18:2n6t	1.30	1.17	1.21	1.22
C18:2n6c	8.50	8.22	8.34	8.35
C18:3n6	16.99	15.54	16.34	15.81
A. glauca				
C14:0	0.50	0.48	0.52	0.48
C16:0	21.21	21.20	22.27	21.94
C16:1	0.57	0.59	0.59	0.59
C18:0 + C18:1n9c	49.40	49.74	48.55	49.39
C18:2n6c	15.06	14.60	15.09	14.61
C18:3n6	10.62	10.23	10.21	9.85
C24:0	0.88	1.16	0.99	1.11
